# PseudoSegRT: efficient pseudo-labelling for intraoperative OCT segmentation

**DOI:** 10.1007/s11548-023-02928-9

**Published:** 2023-05-26

**Authors:** Yu Huang, Riaz Asaria, Danail Stoyanov, Marinko Sarunic, Sophia Bano

**Affiliations:** 1 Department of Computer Science, University College London, London, UK; 2 Wellcome/EPSRC Centre for Interventional and Surgical Sciences (WEISS), University College London, London, UK; 3 Ophthalmology, Royal Free Hospital, London, UK; 4 Institute of Ophthalmology, University College London, London, UK; 5 Department of Medical Physics and Biomedical Engineering, University College London, London, UK

**Keywords:** Deep learning, Pseudo-labelling, Real-time OCT segmentation, Robotic microsurgery, Semi-supervised learning

## Abstract

**Purpose:**

Robotic ophthalmic microsurgery has significant potential to help improve the success of challenging procedures and overcome the physical limitations of the surgeon. Intraoperative optical coherence tomography (iOCT) has been reported for the visualisation of ophthalmic surgical manoeuvres, where deep learning methods can be used for real-time tissue segmentation and surgical tool tracking. However, many of these methods rely heavily on labelled datasets, where producing annotated segmentation datasets is a time-consuming and tedious task.

**Methods:**

To address this challenge, we propose a robust and efficient semi-supervised method for boundary segmentation in retinal OCT to guide a robotic surgical system. The proposed method uses U-Net as the base model and implements a pseudo-labelling strategy which combines the labelled data with unlabelled OCT scans during training. After training, the model is optimised and accelerated with the use of TensorRT.

**Results:**

Compared with fully supervised learning, the pseudo-labelling method can improve the generalisability of the model and show better performance for unseen data from a different distribution using only 2% of labelled training samples. The accelerated GPU inference takes less than 1 millisecond per frame with FP16 precision.

**Conclusion:**

Our approach demonstrates the potential of using pseudo-labelling strategies in real-time OCT segmentation tasks to guide robotic systems. Furthermore, the accelerated GPU inference of our network is highly promising for segmenting OCT images and guiding the position of a surgical tool (e.g. needle) for sub-retinal injections.

## Introduction

**Fig. 1:**
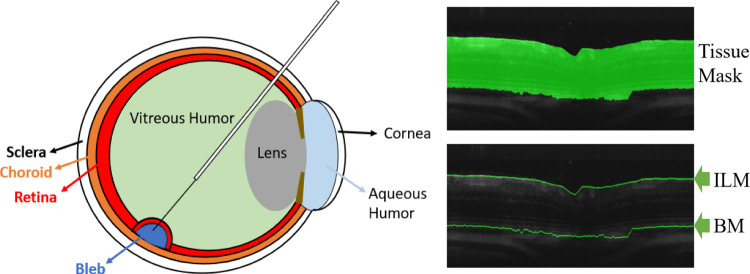
Sub-retinal injection bleb formation (left). A retinal OCT image (right), the top image shows a binary segmentation prediction (Tissue & Background), and the bottom image shows the layer segmentation prediction, where internal limiting membrane (ILM), and Bruch’s membrane (BM) are shown

Optical coherence tomography (OCT) is a non-invasive technique to create high-resolution volumetric images of tissue, and it is a significant technique in the field of ophthalmology [1]. OCT has been widely used in surgeries as it can inform where the surgical instrument is with reference to the sample [2]. Intraoperative OCT imaging can help to enhance the quality of surgical results, and potentially provide guidance for surgical tools [3]. Our ultimate aim is to translate this into real clinical settings where live intraoperative OCT (iOCT) during surgery is displayed for assisting the surgeons. Therefore, we relied on 2D segmentation instead of 3D segmentation as, (a) live iOCT feed displays 2D slices in the operating theatre and the surgeons are familiar with this arrangement, (b) computation of 2D is less expensive than 3D making it better suitable for real-time implementation. For live surgery settings these two factors are essential. Surgery on the eye, especially in the retina, is very challenging due to the delicate tissues [4], and the micrometre-scale manoeuvres. The sub-retinal injection is a way of delivering treatments, which requires a special type of syringe to travel through the entire vitreous humour to the retina. Inside the syringe is a blunt-ended soft-tip cannula that is pushed through the retina, between the photoreceptors (PR) and the retinal pigment epithelium (RPE). This is called the sub-retinal space. Sub-retinal injection to the sub-retinal space is a method shared by emerging treatment approaches such as gene therapy [5]. The sub-retinal space is usually a tight junction. The procedure for gene delivery entails inserting the cannula at this depth in the retina, lifting the retina away from the RPE with a saline solution over the course of 2 to 5 min, creating a bleb, which is a blister-like fluid collection, as shown in Fig. 1, and injecting the viral vector with the new gene.

Collaborative robots are an exciting and rapidly advancing research area with significant potential for improvement in the outcome of micro-surgical procedures. Medical robotics technology is not yet at the level of maturity to significantly reduce the duration of a procedure nor improve patient outcomes compared to a skilled surgeon. An important exception to this comment is sub-retinal injections for gene therapy. Collaborative robotic-assisted sub-retinal injections [6] can be complemented by OCT for positioning the cannula into the sub-retinal space and for cross-sectional visualisation of the bleb. This unique application integrates surgical robotics and medical imaging, where OCT overcomes human limitations by providing micrometre scale resolution imaging of the retina, quantitative measurement of tissue deformation during the creation of the bleb, and improved stability of robot control, thereby enhancing the micro-surgical outcome.

The tasks of surgical robot vision for segmenting the retina and cannula are an important part of sub-retinal injections. Microsurgery requires real-time knowledge of the location of the surgical tool and retinal layers. Machine vision helps provide this information by understanding the scene context through segmentation. OCT layer segmentation in retinal imaging involves dividing the scan into individual retinal layers. In the literature, a variety of techniques are applied for retinal segmentation. Kefieh et al. [7] reviewed the algorithms including pre-processing and OCT segmentation; the commonly used methods for OCT segmentation involve artificial intelligence approaches such as support vector machine [8], active contours approach [9], and graph-based techniques and dynamic programming [10]. However, all these methods rely on huge computations and are often slow. The deep learning method is used to address the problems of traditional OCT segmentation techniques and the computer vision and medical imaging groups have recently invested a lot of effort towards semantic segmentation utilising deep neural networks (DNN). The U-Net architecture, an encoder–decoder design, is proposed by Ronnerberger et al. for biomedical image segmentation [11]. When proper data augmentation and gradient-weighting strategies are applied, a similar architecture can be trained successfully even in a situation that lacks the training data. This architecture shows a satisfactory result for OCT segmentation, and it is adapted into the real-time domain from Borkovina et al.’s work [12]. Another deep neural network called ReLayNet which is implemented by Roy et al. [13] is a fully convolutional neural network. The advantage of ReLayNet is that it not only segments the retinal layers, but it can also segment the fluid (if present), which is not considered in many previous works. DeepRetina proposed by [14], which is adaptive from the Xception network, is another automatic retina layer segmentation framework and also accelerated into the real-time domain but computational performance needed to be improved in future work. Previous works have shown good results for OCT retinal layer segmentation tasks, but most of them are not applicable in real-time situations. Moreover, previous work heavily relies on labelled OCT scans, and a massive amount of unlabelled data is wasted.

Semi-supervised learning (SSL) is a popular technique for utilising large amounts of unlabelled data while minimising the workload on annotators, and a small number of labelled samples are combined with unlabelled samples to carry out a specific task. This area has developed significantly in recent years [2, 15–17]. Entropy minimisation, consistency regularisation, and pseudo-labelling are the three main methods for semi-supervised learning, and most works adhere to one or a combination of these methods. Pseudo-labelling is frequently employed in practice, as it is simple and straightforward [15, 16, 18]. A simple method of training neural networks using pseudo-labelling is proposed by Lee [15] and many pseudo-label strategies are based on this method [16, 19]. It states how to use labelled data and unlabelled data simultaneously to train the model and improve generalisation performance. The theories behind why pseudo-label works are based on are low-density separation between classes and entropy regularisation [15]. Lee’s method relies on a high-confident class and a weight factor which is dependent on the number of epochs. Since it is an efficient way to take advantage of unlabelled data and OCT data intrinsically satisfying the low-density separation principle, we adopted Lee’s method and fit it into OCT segmentation task.

Inspired from Lee [15] approach, we propose *PseudoSegRT*, a simple and efficient approach for real-time pseudo-labelling-based semantic segmentation of iOCT. PseudoSegRT includes a high confidence threshold and a weight function to ensure the accuracy of pseudo-labels and control the contribution of the pseudo-labels during the simultaneous training process. Pseudo-labels update during the training process and become more accurate as the model learns more. Through experimental validation, we demonstrate that PseudoSegRT is suitable for the OCT-related tasks. The trained PSeudoSegRT is then accelerated to a real-time domain to achieve the inference time as little as 1 millisecond. The contributions of the proposed PseudoSegRT include a dynamic weight function that changes the contribution of the pseudo-labels during training, use of only marginal labelled training examples to achieve high performance on unseen test data and real-time implementation making the method suitable for clinical translation and integration in current iOCT settings. We show that the segmentation model (U-Net) is more generalisable with the proposed pseudo-labelling strategy compared to fully supervised learning.

## Proposed method

For the real-time surgical tool guidance and tracking for OCT-guided sub-retinal drug delivery, it is important to detect the ILM (top) and BM (bottom) layers from the OCT (see Fig. 1b. This simplifies the OCT layers segmentation problem to a boundary segmentation problem, i.e. segmenting the OCT image into tissue and background classes.

**Fig. 2:**
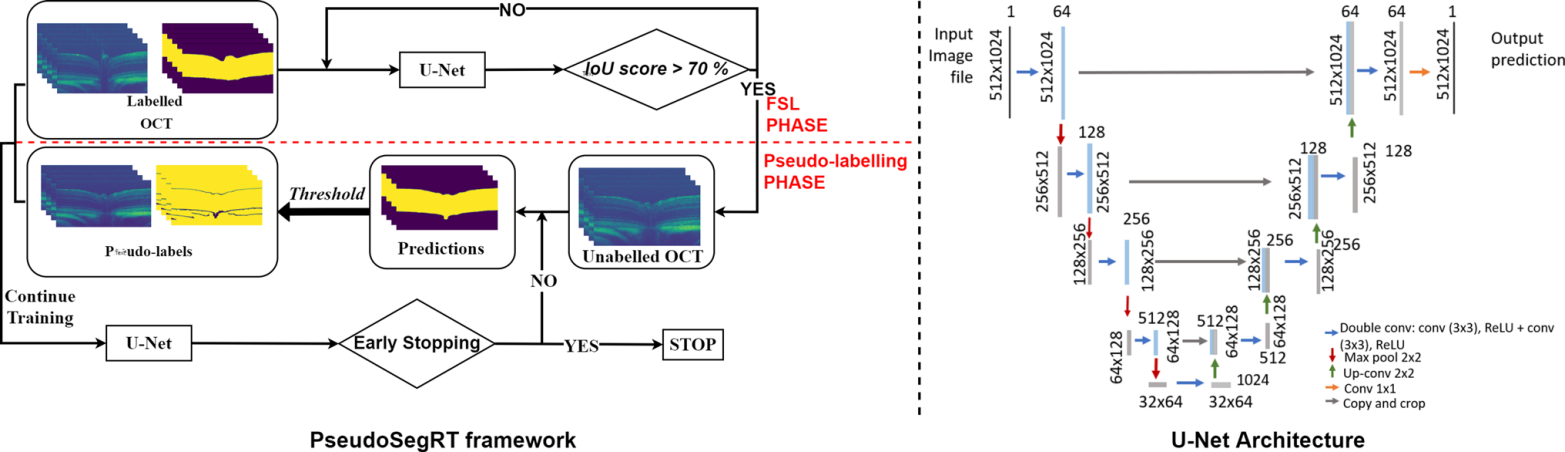
Flowchart of the proposed PseudoSegRT method (left) and diagram of U-Net (right). The proposed strategy first trains a segmentation network in a supervised fashion on the limited dataset. The trained model is used to generate pseudo-labels on limited unlabelled data. The limited labelled and pseudo-labelled data are then used to continue training the segmentation network while updating the pseudo-labels until early stopping is achieved

**Fig. 3:**
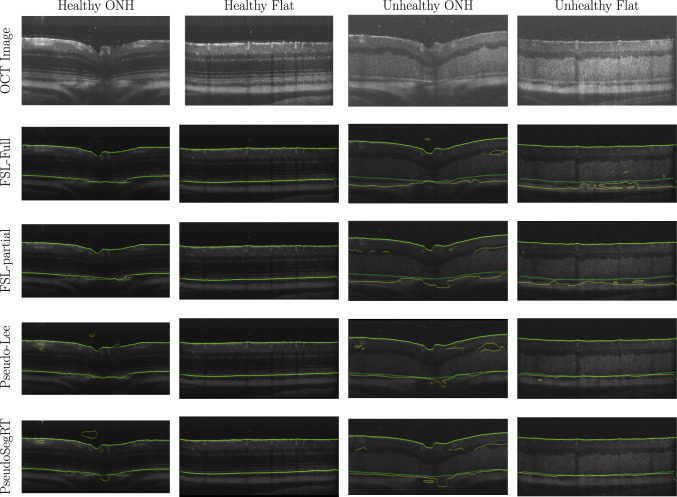
Qualitative comparison of the proposed PseudoSegRT with the fully supervised [11] and Pseudo-Lee [15] methods on unseen healthy and unhealthy scans. Ground-truth tissue boundaries are labelled in green and predicted ones are labelled in yellow

We select U-Net [11] as the base model for the boundary segmentation task, as it has achieved high segmentation accuracy in biomedical image segmentation tasks. U-Net is also shown to be applicable in OCT layer segmentation tasks [12], which is similar to the boundary segmentation task but with more segmentation classes. During the training phase, the model needs to be trained with different patterns of OCT scans (optical nerve head) and flat patterns (shown in Fig. 3). Therefore, labelled data are selected to ensure it captures this variability and different patterns. For the unlabelled data, the OCT scans are randomly chosen. The flowchart of the training process is shown in Fig. 2. The segmentation model is first pre-trained with very limited annotated OCT scans using cross-entropy loss until it reaches the validation intersection over union (IoU) score of over 70%. The pre-trained model is then used to generate pseudo-labels on an equal number of images as that of the labelled training data with a confidence threshold. The pseudo-labels and labelled scans are jointly used to further train the segmentation model. For every epoch, the pseudo-labels are predicted and updated which results in facilitates in improving the pseudo-label confidence. Early stopping is applied to avoid over-fitting.

### Pseudo-segmentation labels

Pseudo-labels are predicted labels for unlabelled data that can be treated as true ground-truth labels. The proposed PseudoSegRT method applies a high-confidence threshold to the prediction mask to obtain the pseudo-labels. During the model’s continued training process, the pseudo-labels are updated and the high-confidence threshold and weight factor of pseudo-label loss are applied. The threshold is set to make sure that every pixel in the pseudo-label can be trusted. Therefore, the high-confident pixels of prediction are selected as pseudo-labels, and the others are ignored. The pseudo-label $y_i$ can be written as:

1 $$\begin{aligned} y_i = {\left\{ \begin{array}{ll} f_{i}(x) = 1 &{}\quad \text {if } f_{i}(x) > 0.9\\ f_{i}(x) = 0 &{}\quad \text {if } f_{i}(x) < 0.1 \\ \text {ignored} &{} \quad \text {otherwise} \end{array}\right. } \end{aligned}$$

where *x* is the unlabelled data and $f_{i}(x)$ is the predicted value at the $i^{th}$ pixel location. The high-confidence threshold is set for two classes: 0 and 1. Only if the confidence of the pixel is higher than 90%, it is used as a pseudo-label giving useful information, and the uncertain pixels will be set to None value, and they will not influence the training of the model.

During the continued training process, binary cross-entropy loss is computed separately for both labelled data ($L_{SL}$) and pseudo-label data ($L_{PL}$). The binary cross-entropy loss is represented as:

2 $$\begin{aligned} L&= l(x,y) = \{l_1, \ldots , l_N\}^T \end{aligned}$$

3 $$\begin{aligned} l_n&= -[y_n \cdot \log x_n + (1 - y_n) \cdot \log (1-x_n)] \end{aligned}$$

where *N* is the batch size, *y* is target, and *x* is prediction.

A weight, $\alpha$, is applied to the pseudo-label loss as a controller to adjust how much we want to rely on the pseudo-labels, and it is used to adjust the influence of the pseudo-label in the combined loss function. $\alpha$ is adaptively computed such that if the pseudo-labels are more accurate, $\alpha$ is higher, thus pseudo-label loss contributes more to the training and vice versa. From the high-confidence selection of pseudo-labels, the accuracy of the pseudo-label can be quantified by the amount of high-confidence prediction. When a higher amount of high-confidence pseudo-labels are generated, they can be trusted more. Therefore, the loss of the pseudo-label can contribute more to the total loss and vice versa. Similarly, it is inversely proportional to the number of pixels that are ignored, which is convenient for calculation. Therefore, the weight function can be expressed as, $\alpha \; \; \; \propto \; \; \; \frac{1}{n_i}$, where $n_i$ represents the number of ignored pixels.

The total loss for training with labelled and unlabelled data simultaneously is then given by:

4 $$\begin{aligned} L = L_{SL} + \alpha \; L_{PL}, \end{aligned}$$

where $L_{SL}$ represents the loss for the labelled data, $L_{PL}$ is the loss of pseudo-labels, and two components of the overall loss function are instances of the previously described function in Eqs. (2) and (3). $\alpha$ is the weight function. We empirically set $\alpha$ to be half of the total number of pixels in the scan divided by the number of pixels ignored.

**Table 1:** Number of OCT scans in the training, validation, test, and unseen test sets

	Training set	Validation set	Test set	Unseen test set
	(Healthy)	(Healthy)	(Healthy)	(Unhealthy)
# scans	1400	300	300	300
# folders	14	3	3	3
# % of background pixels	58	59	57	56
# % of tissue pixels	42	41	43	44

**Table 2:** Comparison of the proposed PseudoSegRT with relevant pseudo-labelling and fully supervised learning methods on the test data

Method	Training data	IoU (%)			PA(%)		
	Used/total	HD	UD	Overall	HD	UD	Overall
Pseudo-Lee [15]	25/1400	95.56	88.26	91.91	98.04	94.41	96.23
Pseudo-notrust	25/1400	94.60	82.55	88.58	97.59	91.54	94.57
FSL-full	1400/1400	**97**.**86**	83.06	90.46	**99**.**06**	91.21	95.14
FSL-partial	5/1400	93.06	81.01	87.04	96.86	90.88	93.87
FSL-partial	25/1400	96.20	80.61	88.41	98.52	90.26	94.39
FSL-partial	50/1400	96.90	77.64	87.27	98.65	89.01	93.83
PseudoSegRT	5/1400	92.20	81.36	86.78	96.49	91.30	93.90
PseudoSegRT	25/1400	96.19	**88**.**88**	**92**.**54**	98.31	**94**.**47**	**96**.**39**
PseudoSegRT	50/1400	97.27	85.75	91.51	98.80	93.26	96.03

Key: HD, healthy dataset; UD, unhealthy dataset; IoU, mean intersection over union; PA, average pixel accuracy

### Model acceleration for real-time deployment

The resolution of the data used is too high, as the resolution of the OCT scans is 1000$\times$575 pixels, and the CPU computational power is unable to deal with the data size. As NVIDIA GPU gives powerful computational capability, U-Net can be optimised using TensorRT. The pipeline to convert the PyTorch model is to: convert the PyTorch model to the ONNX^1^ model, and then to the TensorRT engine. When converting the ONNX model to the TensorRT model, the precision of the engine should be chosen. Although neural network parameters are trained using 32-bit floating point (FP32), TensorRT offers support for FP32 (32-bit floating point), FP16 (16-bit floating point), and INT8 (8-bit integer) precision modes. The inference time may be significantly reduced by selecting a lower precision option. It must be ensured that the performance of the model should still be satisfying with a high speed.

### Dataset description and preparation

The dataset used for boundary segmentation is from a public data set [20], and it uses spectral domain optical coherence tomography (SD-OCT) scans from mouse models. SD-OCT scans use the spectral detection method for OCT, which interrogates all depths at once and can give a faster speed relative to the time-domain OCT. In this data set, SD-OCT scans were acquired from ten mice, five mice that were kept as healthy controls, and an additional five mice imaged on four days—1, 3, 6, and 9 days after light-induced retinal damage. A rectangular scanning mode is used in this data set, and ten B-scans from ten locations are obtained from SD-OCT ophthalmic imaging system. In this paper, the healthy data set is used for the training process. The healthy data set contains 2000 OCT scans from five mice acquired on four different days, and the data set is separated into the training, validation, and test sets with a ratio of (7: 1.5: 1.5) as mentioned in Table 1. None of the unhealthy scans are used in the training process which is mainly used as testing the generalisation capabilities of the proposed method.

Original labelled data provides information about eight retinal layers [20] but the boundary segmentation task requires binary segmentation (only tissue and background). Thus, a binary mask was applied to segment eight retinal layers (from ILM to BM—see Fig. 1) as tissue and the other area as background. For the model training, all volumes were resized to 512 $\times$1024 pixels and pre-processed for boundary segmentation labels.

**Table 3:** Comparison of the proposed PseudoSegRT (reporting mean intersection over union) with various values of confidence threshold when using 25 labelled scans in training

Confidence threshold	Healthy dataset	Unhealthy dataset	Overall
70%	96.02	87.10	91.56
80%	**96**.**36**	87.66	92.01
90%	96.19	**88**.**88**	**92**.**54**

## Experimental setup

### Training details

All experiments are performed using a Linux machine having an NVIDIA GTX Titan X card with a memory of 12 GB and an Intel(R) Xeon(R) CPU E5-2620 v3, at 2.40 GHz. The PyTorch framework is used for the model implementation. As the input data are in grayscale format, and the prediction is also in a single channel, 1–1 binary U-Net is used as shown in Fig. 2. Models are trained using Adam optimiser with the learning rate of $10^{-5}$. The batch size is set to 1 due to GPU memory limit. The validation IoU is set to 70% for pre-training (FSL phase). The total number of epochs for pseudo-labelling training is set to 40 epochs with early stopping applied with patience of 10 epochs. This early stopping criterion terminates the training process when the validation IoU score remains less than the maximum recorded IoU for continuous 10 epochs. The weights captured at the maximum IoU are used during model testing.

### Data augmentation

Data augmentation is a technique to apply slight modifications to existing data and obtain new data to train the model, which is efficient to expand the diversity of the existing data set. Considering the characteristics of the OCT scans, in these experiments, random rotation (angle between 10 and $20^{\circ }$) and a change of brightness factor (1–2) were applied during the training process.

### Evaluation metric

The Metric used to evaluate the performance of the model is the mean IoU score and the mean Pixel Accuracy (PA), since these are the most commonly used metric for segmentation. IoU measures the degree of overlap between the ground-truth and predicted segmentation masks. As the amount of the background class and tissue class are balanced (57.16% background and 42.84% tissue), pixel accuracy is chosen which measures the percentage of the correctly classified pixels.

### Comparison methods

To evaluate the performance of the proposed PseudoSegRT, we perform its comparison with (1) FSL-full: the fully supervised learning method trained on the full training set of 1400 OCT scans, (2) FSL-partial: the fully supervised learning method trained only using 25 training images, (3) Pseudo-Lee: the pseudo-labelling method proposed by Lee [15], and (4) Pseudo-notrust: our proposed pseudo-labelling approach without the confidence threshold and weight function. In the case of the pseudo-labelling approaches, 25 labelled OCT scans and an equal number of pseudo-labelled OCT scans are used during the training process.

### Ablation study

To understand how many training and pseudo-labelled samples are required to achieve comparable performance as that of a fully supervised approach, we perform ablation experiments. We trained PseudoSegRT with 5, 10,..., 60 labelled and an equal number of unlabelled scans. Likewise, we trained FSL-partial with 5, 10,..., 60 scans. For simplicity, we only report the performance on 5, 25, and 50 scans in Table 2. We can observe from this table that the two models converged at 25 scans dataset, with PseudoSegRT even surpassing FSL-full in unhealthy test data. Beyond 25 scans, there was no significant performance gain. Therefore, we selected 25 scans as the training set for the rest of the pseudo-labelling experiments.

**Table 4:** Acceleration result: comparison between raw PyTorch model, accelerated TensorRT Engine with precision FP16 and TensorRT Engine with precision FP32

	IoU score	Inference time	Frames/second
	(%)	(ms)	(fps)
PyTorch model	96.19	493.37	2
TensorRT Engine (FP32)	95.78	3.14	318.5
TensorRT Engine (FP16)	95.78	0.67	1492.5

## Results and discussion

Quantitative comparisons of fully supervised learning and pseudo-labelling methods are presented in Table 2. It can be observed that though FSL-full achieved the best performance on the healthy test dataset, it failed to generalise on the unseen unhealthy test dataset. As shown in Fig. 3, the unseen unhealthy dataset has large difference compared with the healthy dataset; as a result FSL methods failed in such situation. Likewise, the performance of FSL-partial was comparatively low. All pseudo-labelling techniques achieved better performance, showing better generalisation capabilities compared to the FSL approaches. The proposed PseudoSegRT outperformed Lee’s method in both the healthy dataset and the unhealthy dataset giving an overall IoU of 92.54%.

The qualitative results for model comparison are presented in Fig. 3. Compared to FSL-full, our proposed method and Lee’s method shows better generalisability and are more competitive for unseen data even when a limited amount of training data is applied. This result shows the limitation of fully supervised learning, and it is useful to apply a pseudo-labelling strategy to deal with unseen data. From Fig. 3, it can be seen that the fully supervised learning method showed good performance on the healthy samples (row 1–2), but it almost failed to detect the bottom boundary of the unseen data (row 3–6). Lee’s method and our proposed method sacrifice a little amount of accuracy of the data which has similar distribution learned but achieve improved generalisability. With our proposed PseudoSegRT method, the pseudo-labels can give better performance by applying the weight factor design and updating the pseudo-labels during training. The pseudo-labelling method which does not have a threshold and weight function gave a worse performance compared with other pseudo-labelling methods. Pseudo-labels without a high confidence threshold may disturb the training process as the information of the unlabelled data is not used efficiently as results shown in Table 3. From Table 3, it can be observed that the overall performance of the model increases with the value of the confidence threshold.

As the clean OCT scans satisfy the low-density separation, the results of the proposed method show a good performance for layer segmentation. However, it requires further tests for complicated situations, such as when a surgical tool appears in the OCT scan. Further study can also be done for surgical tool detection.

Following the details mentioned in section “Model acceleration for real-time deployment”, we accelerate the segmentation model using TensorRT. The inference time (in milliseconds) and processing frequency (in frames per second) are reported in Table 4. The FP16 implementation on TensorRT came out to be extremely efficient with an inference time of only 0.67 milliseconds. Improved generalisability and increased efficiency make the proposed PseudoSegRT to be suitable for intraoperative OCT segmentation.

## Conclusion

We proposed a simple and efficient pseudo-labelling approach for semi-supervised segmentation in intraoperative optical coherence tomography (iOCT). We showed the significance of obtaining high-confidence pseudo-labels through high-confidence thresholding and adaptative weighting during training. We showed that PseudoSegRT is suitable for the application of OCT-related tasks. Sometimes, there are a limited amount of manual segmentation provided, and a pseudo-labelling strategy can make use of the unlabelled data set. The experimentation with pseudo-labelling on the mouse retina data had results that were comparable to the fully supervised performance on the original test set. The benefit of the pseudo-labelling was demonstrated on the additional test set of diseased eyes, which suggested that the additional (pseudo-labelled) image data used for training helped to improve the generalisability of the neural network on out-of-distribution images. Therefore, a robust and generalisable neural network can be created which has the potential to function correctly even in the presence of unseen data. The OCT segmentation task can be accelerated into a real-time domain. From the result of the boundary segmentation, more than 1400 frames can be segmented in a second efficiently. The future work will focus on post-processing to filter out the noisy predictions and integration of the method in the in laboratory setup for OCT-guided robotic microsurgery.

## Footnotes

1 https://onnx.ai/.

## References

[1] Bhende M, Shetty S, Parthasarathy MK, Ramya S (2018). Optical coherence tomography: a guide to interpretation of common macular diseases. Indian J Ophthalmol.

[2] Seibold CM, Reiß S, Kleesiek J, Stiefelhagen R (2022) Reference-guided pseudo-label generation for medical semantic segmentation. In: Proceedings of the AAAI conference on artificial intelligence, vol 36, pp 2171–2179

[3] Carrasco-Zevallos OM, Keller B, Viehland C, Shen L, Seider MI, Izatt JA, Toth CA (2016). Optical coherence tomography for retinal surgery: perioperative analysis to real-time four-dimensional image-guided surgery. Invest Ophthalmol Vis Sci.

[4] Gupta PK, Jensen PS, de Juan E (1999) Surgical forces and tactile perception during retinal microsurgery. In: Medical image computing and computer-assisted intervention—MICCAI’99: second international conference, Cambridge, UK, September 19-22, 1999. Proceedings 2, pp. 1218–1225. Springer

[5] Xue K, Groppe M, Salvetti A, MacLaren R (2017). Technique of retinal gene therapy: delivery of viral vector into the subretinal space. Eye.

[6] Gerber MJ, Pettenkofer M, Hubschman J-P (2020). Advanced robotic surgical systems in ophthalmology. Eye.

[7] Kafieh R, Rabbani H, Kermani S (2013). A review of algorithms for segmentation of optical coherence tomography from retina. J Med Signals Sens.

[8] Zawadzki RJ, Fuller AR, Wiley DF, Hamann B, Choi SS, Werner JS (2007). Adaptation of a support vector machine algorithm for segmentation and visualization of retinal structures in volumetric optical coherence tomography data sets. J Biomed Opt.

[9] Yazdanpanah A, Hamarneh G, Smith BR, Sarunic MV (2010) Segmentation of intra-retinal layers from optical coherence tomography images using an active contour approach. IEEE Trans Med Imaging 30(2):484–496 10.1109/TMI.2010.2087390 20952331

[10] Yang Q, Reisman CA, Wang Z, Fukuma Y, Hangai M, Yoshimura N, Tomidokoro A, Araie M, Raza AS, Hood DC (2010). Automated layer segmentation of macular oct images using dual-scale gradient information. Opt Express.

[11] Ronneberger O, Fischer P, Brox T (2015). U-net: convolutional networks for biomedical image segmentation.

[12] Borkovkina S, Camino A, Janpongsri W, Sarunic MV, Jian Y (2020). Real-time retinal layer segmentation of OCT volumes with GPU accelerated inferencing using a compressed, low-latency neural network. Biomed Opt Express.

[13] Roy AG, Conjeti S, Karri SPK, Sheet D, Katouzian A, Wachinger C, Navab N (2017). Relaynet: retinal layer and fluid segmentation of macular optical coherence tomography using fully convolutional networks. Biomed Opt Express.

[14] Li Q, Li S, He Z, Guan H, Chen R, Xu Y, Wang T, Qi S, Mei J, Wang W (2020). Deepretina: layer segmentation of retina in oct images using deep learning. Transl Vis Sci Technol.

[15] Dong-Hyun L (2013) Pseudo-label: The simple and efficient semi-supervised learning method for deep neural networks. In: Workshop on challenges in representation learning, ICML vol 3, p 896

[16] Renz K, Stache NC, Fox N, Varol G, Albanie S (2021) Sign segmentation with changepoint-modulated pseudo-labelling, pp 3403–3412

[17] Thompson BH, Di Caterina G, Voisey JP (2022) Pseudo-label refinement using superpixels for semi-supervised brain tumour segmentation, pp 1–5. IEEE

[18] Dopierre T, Gravier C, Subercaze J, Logerais W (2020) Few-shot pseudo-labeling for intent detection. In: Proceedings of the 28th international conference on computational linguistics, pp 4993–5003

[19] Cascante-Bonilla P, Tan F, Qi Y, Ordonez V (2021) Curriculum labeling: Revisiting pseudo-labeling for semi-supervised learning. In: Proceedings of the AAAI conference on artificial intelligence, vol 35, pp 6912–6920

[20] Antony BJ, Kim B-J, Lang A, Carass A, Prince JL, Zack DJ (2017). Automated segmentation of mouse OCT volumes (ASiMOV): Validation and clinical study of a light damage model. PLoS ONE.

